# Exposure of Wild Ungulates to the Usutu and Tick-Borne Encephalitis Viruses in France in 2009–2014: Evidence of Undetected Flavivirus Circulation a Decade Ago

**DOI:** 10.3390/v12010010

**Published:** 2019-12-19

**Authors:** Laure Bournez, Gérald Umhang, Eva Faure, Jean-Marc Boucher, Franck Boué, Elsa Jourdain, Mathieu Sarasa, Francisco Llorente, Miguel A. Jiménez-Clavero, Sara Moutailler, Sandrine A. Lacour, Sylvie Lecollinet, Cécile Beck

**Affiliations:** 1 Nancy Laboratory for Rabies and Wildlife, The French Agency for Food, Environmental and Occupational Health and Safety (ANSES), CS 40009 54220 Malzéville, France; gerald.umhang@anses.fr (G.U.); jean-marc.boucher@anses.fr (J.-M.B.); franck.boue@anses.fr (F.B.); 2 National Hunters Federation, 92130 Issy-les-Moulineaux, France; efaure@chasseurdefrance.com (E.F.); msarasa@beops.fr (M.S.); 3 Université Clermont Auvergne, INRAE, VetAgro Sup, Unité mixte de recherche Epidémiologie des maladies animales et zoonotiques (UMR EPIA), 63122 Saint-Genès-Champanelle, France; elsa.jourdain@inra.fr; 4 Biologie et Ecologie des Organismes et Populations Sauvages (BEOPS), 1 Esplanade Compans Caffarelli, 31000 Toulouse, France; 5 Centro de Investigación en Sanidad Animal, Instituto Nacional de Investigación y Tecnología Agraria y Alimentaria (INIA-CISA), 28130 Valdeolmos, Spain; dgracia@inia.es (F.L.); majimenez@inia.es (M.A.J.-C.); 6 Centro de Investigación Biomédica en Red de Epidemiología y Salud Pública (CIBERESP), 28029 Madrid, Spain; 7 Unité mixte de recherche Biologie moléculaire et Immunologie Parasitaire (UMR BIPAR), ANSES, INRAE, Ecole Nationale Vétérinaire d’Alfort, Université Paris-Est, Maisons-Alfort 94700, France; sara.moutailler@anses.fr; 8 Unité mixte de recherche (UMR) Virologie, INRAE, Ecole Nationale Vétérinaire d’Alfort, ANSES, Université Paris-Est, 94700 Maisons-Alfort, France; sandrine.lacour@anses.fr (S.A.L.); sylvie.lecollinet@anses.fr (S.L.); cecile.beck@anses.fr (C.B.)

**Keywords:** flavivirus, usutu virus, tick-borne encephalitis virus, west nile virus, seroprevalence, wild ruminants, roe deer, wild boar

## Abstract

Flaviviruses have become increasingly important pathogens in Europe over the past few decades. A better understanding of the spatiotemporal distribution of flaviviruses in France is needed to better define risk areas and to gain knowledge of the dynamics of virus transmission cycles. Serum samples from 1014 wild boar and 758 roe deer from 16 departments (administrative units) in France collected from 2009 to 2014 were screened for flavivirus antibodies using a competitive ELISA (cELISA) technique. Serum samples found to be positive or doubtful by cELISA were then tested for antibodies directed against West Nile virus (WNV), Usutu virus (USUV), Bagaza virus (BAGV), and tick-borne encephalitis/Louping ill viruses (TBEV/LIV) by microsphere immunoassays (except BAGV) and micro-neutralization tests. USUV antibodies were detected only in southeastern and southwestern areas. TBEV/LIV antibodies were detected in serum samples from eastern, southwestern and northern departments. The results indicate continuous circulation of USUV in southern France from 2009 to 2014, which was unnoticed by the French monitoring system for bird mortality. The findings also confirm wider distribution of TBEV in the eastern part of the country than of human clinical cases. However, further studies are needed to determine the tick-borne flavivirus responsible for the seroconversion in southwestern and northern France.

## 1. Introduction

The importance of the *Flavivirus* genus (family *Flaviviridae*) in terms of public and animal health has increased in Europe over the last few decades [1,2,3]. Many of these viruses, such as West Nile virus (WNV) or tick-borne encephalitis virus (TBEV), are major human pathogens. In nature, these viruses are maintained in an enzootic cycle involving ornithophilic mosquitoes (WNV, Usutu virus, and Bagaza virus) or ticks (TBEV and Louping ill virus) as competent vectors, and birds or small mammals as main reservoir hosts. Most medium and large mammalian species are susceptible to infections, but are considered dead-end or incidental hosts as they are not able to transmit the viruses. Louping ill virus (LIV) is an exception, as sheep, hares and grouse are considered to be the main reservoirs [4].

In recent decades, vector-borne flavivirus infections have emerged in new areas worldwide, and there have been continuous and growing reports of outbreaks in humans or animals [3,5,6]. In Western Europe, an increase in the number of WNV cases in horses and humans and of TBEV cases in humans has been reported broadly, while both viruses have been spreading to previously unaffected areas [3,5,6]. In addition, the Usutu virus (USUV), a virus belonging to the Japanese encephalitis complex, like WNV, was detected for the first time in Italy in 1996 and in Austria in 2001, causing deaths among Eurasian blackbirds (*Turdus merula*) [7]. In the following years, the USUV genome or antibodies were found in several Central and Western European countries including Austria, Spain, Hungary, Belgium, the Czech Republic, Germany, Poland, Greece, France, Croatia, Serbia, Slovakia, Switzerland and the Netherlands, in areas that frequently overlapped with WNV circulation zones [2,8]. Two major USUV outbreaks affecting wild birds were identified with extensive circulation in Northern Europe in 2016 (Belgium, Germany, the Netherlands and France) [9], and in Western and Central Europe in 2018 (France, Austria) [2,10]. Given that USUV and WNV are genetically, antigenically and epidemiologically closely related, in Europe the question emerged of the influence of one virus on the spatiotemporal dynamics of the other. This highlights the need to obtain more epidemiological data on the spatiotemporal dynamics of USUV in endemic and nonendemic areas of WNV. 

A better understanding of the spatiotemporal distribution of flaviviruses is needed to define risk areas of virus circulation, and to gain knowledge on the dynamics of virus transmission cycles. Although global changes (climatic, environmental or anthropogenic factors) may impact the geographical distribution of tick-borne flaviviruses, these viruses generally exhibit more epidemiological stability over time than mosquito-borne flaviviruses, which evolve by epizootic waves [6]. WNV, USUV and TBEV are the main flaviviruses that have been circulating in France. Two other seabird-related tick-borne flaviviruses have occasionally been found on the Atlantic coast: Meaban virus and Tyuleniy virus [6]. The presence of Louping ill virus (LIV) or Louping ill-like virus (LI-like virus) and Bagaza virus (BAGV) in neighbouring countries, including Spain [6], raises questions about the potentially undetected circulation of these flaviviruses in France. The endemic area for WNV in France is located along the Mediterranean coast, more specifically in the Camargue region, a natural wetland. However, the virus has been detected irregularly during the past two decades. WNV epizootics with neuroinvasive cases in horses were reported in France in 2000, 2003, 2004, 2006, 2015 and 2018‒2019 [11,12,13]. Between these epidemic years, it is unknown whether the virus circulated every year in some areas, or whether epizootics are caused by new strains introduced by migrating birds, even though the identification of silent circulation episodes by active surveillance and virus genome analysis tend to support the first hypothesis [14,15]. The presence of USUV was suspected in 2009–2010 in Camargue, with six Eurasian magpies (*Pica pica*) displaying USUV-specific neutralizing antibodies [15]. Since then, several lineages of the virus have been detected from 2015 to 2018 in several French regions from dead birds, one human patient or mosquito pools [13,16,17,18,19], but there is no information on the spatiotemporal circulation of USUV in France between 2009 and 2015. 

France is located in the western area of the TBEV geographical distribution. Compared to neighbouring countries (e.g., Switzerland and Germany), the number of human TBEV cases is relatively stable, from two to 29 per year [3]. Clinical cases in humans have only been reported in some areas of eastern France: Mainly in Alsace-Lorraine, in the Alpine area (Haute-Savoie), and recently in the Loire and Haute-Loire [20,21]. Although human infection seems to be restricted to certain areas, the virus is thought to circulate to a larger extent in eastern France. In fact, TBEV-positive serum samples from forest workers from other regions of eastern France (Bourgogne, Franche-Comté and Ardennes) have been detected [22]. Nevertheless, as it is often difficult to know the exact location where people were infected through the bites of TBEV-infected ticks, these results require further investigation to better define the areas where TBEV circulates. Other viruses antigenically closely related to TBEV belonging to the TBEV serocomplex, LIV and LI-like viruses, such as Turkish sheep encephalomyelitis (TSE) and Spanish sheep encephalomyelitis (SSE), are well known in the British Isles and Ireland, but have also been detected sporadically in northern Spain (Basque Country, Asturias), Norway, Denmark, Turkey, Bulgaria and Greece [23,24,25,26,27]. In these countries, except for Great Britain (LIV), data on the natural transmission cycle are not available. Recently, TBEV serocomplex neutralizing antibodies were detected in dogs in the south of Spain [28] and in horses on the island of Mallorca (off the eastern coast of Spain) [29]. Based on our current knowledge of the TBEV epidemiological cycle, it is more likely that the virus involved is LIV or a LI-like virus. The key factor in TBEV persistence in a given area is thought to be co-feeding of tick larvae and nymphs. These stages have synchronous activities only under specific climatic conditions that are not found in Spain [30]. 

Hunter-harvested mammals are more accessible than wild birds or small mammals and may be used as sentinels for monitoring flavivirus activity [31,32,33]. Antibodies against WNV, USUV and TBEV have been detected in naturally exposed roe deer (*Capreolus capreolus*), red deer (*Cervus elaphus*) and wild boar (*Sus scrofa*) [31,32,34,35,36]. These species have a long life span and antibodies against flaviviruses may persist for more than one year [31,32,34,35,37]. Thus, analysing serum from all age classes is useful to explore the spatiotemporal flavivirus trends in a given area. In this study, we used serum banking of samples from hunter-harvested wildlife to estimate the exposure level of wild boar and roe deer to flaviviruses in several regions of France from 2009 to 2014. The main objectives were: 1) to improve knowledge on TBEV distribution; 2) to determine whether LIV or BAGV are present in France; and 3) to determine whether USUV and WNV circulated from 2009 to 2014 and if so, in which areas. 

## 2. Materials and Methods 

### 2.1. Data and Study Area

**Figure 1:**
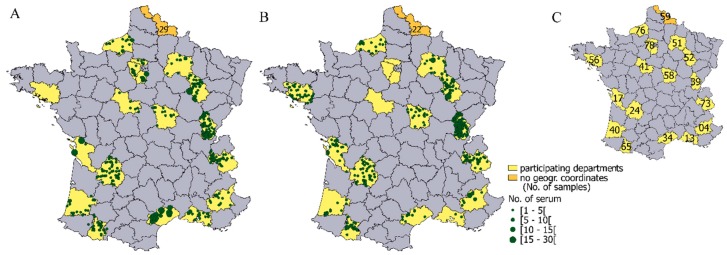
Geographical distribution of the serum samples from wild boar (**A**) and roe deer (**B**), analysed for the detection of flavivirus antibodies using competitive ELISA. The size of each point is defined according to the number of serum samples per municipality. In department 59, as the geographical coordinates associated with the collected serum samples were not available, we indicated the number of serum samples sampled within the department. In (**C**), the numbers represent the department code.

In 2009, the French National Hunter’s Federation launched a voluntary program for departmental (French administrative unit) hunting associations aimed at serum banking of wild ungulate samples. Blood samples were taken from the thoracic cavity of freshly killed hunted animals and centrifuged. Serum samples were then stored at −20 °C by local hunter’s associations. Animals were killed during the hunting season, which is from September to February. Animals were classified by gender and by age according to dentition patterns (juveniles: <1 year old and adults: >1 year old). Among the 31 departments in which local hunter’s associations participated in this program, we selected 18 departments to represent different bioregions of France (Figure 1C). For the sampling scheme and analysis, given their smaller size, the departments from the “Ile-de-France” region (departments 78, 91, and 95 in Figure 1C) were considered one department. We used all serum samples collected from these departments from 2009 to 2014. 

### 2.2. Laboratory Analysis

Antibodies against flaviviruses were detected using a commercial competitive enzyme-linked immunosorbent assay (cELISA; ID Screen^®^ West Nile Competition, ID Vet, Montpellier, France) based on purified whole WNV antigen for the detection of antibodies directed against the envelope E protein common to all flaviviruses. Although this ELISA test is designed for WNV detection, the high cross-reactivity observed makes this method suitable for the detection of a broad range of flavivirus antibodies [38,39]. The test was performed according to the manufacturer’s instructions and interpreted following their recommendations: Samples with a sample-to-negative control (S/N) ratio ≤40%, [40–50%] and >50% were considered positive, doubtful and negative, respectively. 

To identify the virus causing the seroconversion, samples with positive or doubtful results were then tested for the presence of antibodies against WNV, USUV, TBEV, and LIV by two methods, xMAP microsphere immunoassays (MIAs) and virus micro-neutralization tests (MNTs), as detailed below. In addition, these samples were also tested for BAGV antibodies by virus micro-neutralization. 

Serological screening of samples was performed using MIA technology. Purified recombinant envelope domain III (rEDIII) proteins of WNV, USUV, TBEV and LIV were used for the capture of specific IgG antibodies. A recombinant soluble ectodomain of WNV envelope (E) glycoprotein (WNV.sE), which identifies infection by all flaviviruses in the same way as the competitive ELISA test, and WNV.EDIII, USUV.EDIII, TBEV.EDIII and LIV.EDIII were coupled to MagPlex^®^ Microspheres using a Bio-Plex Amine Coupling Kit (Bio-Rad Laboratories, Hercules, CA, USA), according to the manufacturer’s instructions, as described in Beck et al. (2015) [39]. Five micrograms of WNV.sE, TBEV.EDIII or LIV.EDIII or 50 µg of WNV.EDIII and USUV.EDIII were coupled to the beads. The serum samples (diluted 1:100) were then tested with MIA technology using an equimolar mixture of the five beads, as previously described in Beck et al. (2015) [39]. The secondary antibody was a rabbit anti-deer IgG biotin conjugate (Interchim) for roe deer serum samples, and a goat anti-pig IgG biotin conjugate for wild boar serum samples (AB_2338429, Jackson Immunoresearch, West Grove, PA, USA), diluted 1:500. For each antigen, the diagnostic cutoff was determined from the mean of median of fluorescence (MFI) values of 25 pig (for the analysis of wild boar serum samples) or roe deer (for the analysis of roe deer serum samples) negative serum samples plus three standard deviations of the mean. The 25 pig and roe deer serum samples used to determine the cutoff were sampled in the field from several departments of France and were found to be negative by cELISA. In cases of positive reactions with several rEDIIIs for viruses belonging to the Japanese (i.e., USUV and WNV) or tick-borne encephalitis serocomplexes (i.e., TBEV and LIV), an animal was considered infected with a specific flavivirus if the corresponding bead coupled to rEDIII generated an MFI at least double that generated with the other beads.

MNTs were performed for the detection of specific neutralizing antibodies against WNV (strain Is98, Genbank ID AF481864.1, provided by Philippe Desprès, IPP, Paris), USUV (strain Italy 2012, 206795-3/2012, Genbank ID KX816653.1, provided by Davide Lelli, IZSLER, Brescia), TBEV (strain Hypr, Genbank ID U39292.1), LIV (strain Li 3/1, Genbank ID KP144331.1, provided by Nicholas Johnson, APHA, Weybridge), and BAGV (strain Spain 2010, Genbank KR108244) as described in Beck et al. (2015) [39]. A serum sample was considered positive if cells were protected at the 1:10 serum dilution for WNV, USUV and BAGV, and 1:20 for TBEV and LIV. When results indicated cross-neutralization between flaviviruses, we identified the infecting flavivirus by considering the virus with the highest neutralization capacity, and with neutralization titres that differ by at least 4-fold. 

### 2.3. Laboratory Result Interpretation

Detection of flaviviruses. We considered a serum sample to be i) “flavivirus-negative” when the result of the pan-flavivirus cELISA was negative or was doubtful and negative in MNTs or MIAs; or ii) “flavivirus-positive” when flavivirus antibodies were detected by the pan-flavivirus cELISA (positive serum in cELISA), MNTs or MIAs (doubtful serum in cELISA). 

Identification of flaviviruses. As MNTs and MIAs gave complementary and specific results and identified the same viruses (see Results 3.1), we considered that a serum sample was positive for a specific virus (i.e., USUV-, TBEV-, LIV-, WNV- or BAGV-positive) when antibodies towards this virus were detected by at least one of the methods, MNTs or MIAs, given the conditions described above. TBEV and LIV could not be differentiated by MNTs and MIAs, and hereafter results are noted as “TBEV/LIV positive.” 

We considered a positive or doubtful cELISA serum sample as “confirmed” when the flavivirus responsible for the seroconversion was identified by one of the confirmatory tests, MNTs or MIAs. Positive cELISA serum samples not identified by MNTs or MIAs were considered “non-confirmed”. We calculated the proportion of confirmatory results as the proportion of confirmed serum samples among positive or doubtful cELISA serum samples. We compared the S/N ratio of the cELISA between serum samples confirmed by two or only one test to assess whether the difference in the results was correlated to the S/N ratio. 

### 2.4. Statistical Analyses 

The apparent seroprevalence of antibodies against flaviviruses was estimated by species from the ratio of cELISA positives to the total number of samples, with the exact binomial confidence intervals of 95% (CI_95%_). Differences between species were tested by a Pearson’s Chi-square test. The mean cELISA S/N ratio values were compared between species by a Student’s *t*-test. 

As no antibodies were detected against WNV and BAGV, we only analysed the factors associated with USUV and TBEV/LIV antibody seroprevalence. This analysis was only conducted in USUV- and TBEV/LIV-infected departments. A department was considered USUV-infected (or TBEV/LIV-infected) when at least one serum sample was USUV- (or TBEV/LIV-) positive in MNT or MIA. For TBEV/LIV, we only included the departments of eastern France, the known geographical distribution area of the TBE virus. We considered that the TBEV/LIV-positive serum samples from eastern France were likely “TBEV-positive.” In infected departments, the exposure status of animals for each virus was modelled using binomial generalized linear mixed model (GLMM), as a function of sampling period, ungulate species and age. The department was included as a random factor to take into account the potential spatial aggregation in virus circulation. We defined the sampling period from 1 September to 28 February of the following year, in order to take into account the hunting season and the probable low flavivirus circulation during the winter. Odds ratios and 95% confidence intervals were estimated by bootstrapping. All statistical analyses were performed with R 3.5.0 software (Vienna, Austria) [40]. All maps were created with QGIS 2.18.11 (QGIS Geographic Information System. Open Source Geospatial Foundation Project).

## 3. Results

### 3.1. Serological Results

In total, 758 roe deer serum samples and 1014 wild boar serum samples were collected from September 2009 to November 2014 (Figure 1). From eight to 304 serum samples were collected per department (Figure 1 and Table 1). Pan-flavivirus antibodies (i.e., positive and doubtful cELISA results) were detected in 16/758 (2.1%, CI_95%_: 1.2‒3.4%) roe deer serum samples and 57/1014 (5.6%, CI_95%_: 4.3‒7.2%) wild boar serum samples. These data represent a significantly higher seroprevalence in wild boar than in roe deer (Chi^2^ *p* < 0.001). Among positive pan-flavivirus cELISA samples, the mean % S/N value of wild boar serum samples was lower than that of roe deer (Student’s *t*-test, *t* = −3.7, df = 29, *p* < 0.001), suggestive of a stronger antibody response in wild boar.

Among the ELISA positive and doubtful samples (*n* = 73), seven could not be tested by MNTs due to serum cytotoxicity or low serum quantity (Table 1). None of the serum samples tested contained specific antibodies against WNV or BAGV. Specific antibodies against USUV or TBEV/LIV were detected in 32 and four serum samples by MNTs, and in 30 and seven serum samples by MIAs, respectively. The results were identical between both methods for 49 serum samples (Table 2): 22 were negative, 26 positive for USUV, and one positive for TBEV/LIV, with a higher proportion of identical results among the USUV-positive serum samples (26/35, 74%) than the TBEV/LIV-positive serum samples (1/9, 11%). The mean % S/N value was lower for samples confirmed by MNT and MIA than that confirmed by only one test, or that not confirmed by any test (Mann–Whitney U test, W = 330, *p* = 0.007), suggesting that high antibody levels facilitated the confirmation of flavivirus infection.

Overall, specific antibodies against USUV were detected by either method in two roe deer and 34 wild boar serum samples, and those against TBEV/LIV were detected in one roe deer and nine wild boar serum samples. Neutralizing antibody titres were around 1/10–1/20 for 11 samples and ranged from 1/40 to 1/320 for 23 samples. The mean % S/N value was positively correlated with the neutralizing antibody titres (Spearman correlation, r_s_ = 0.88, *p*-value = 0.03), suggesting again that high antibody levels facilitated the confirmation of flavivirus infection by MNT. The proportion of confirmatory results (i.e., positive for at least one test) was higher in wild boar (43/57, 75%) than in roe deer (2/16, 13%). 

### 3.2. Geographical Distribution

**Figure 2:**
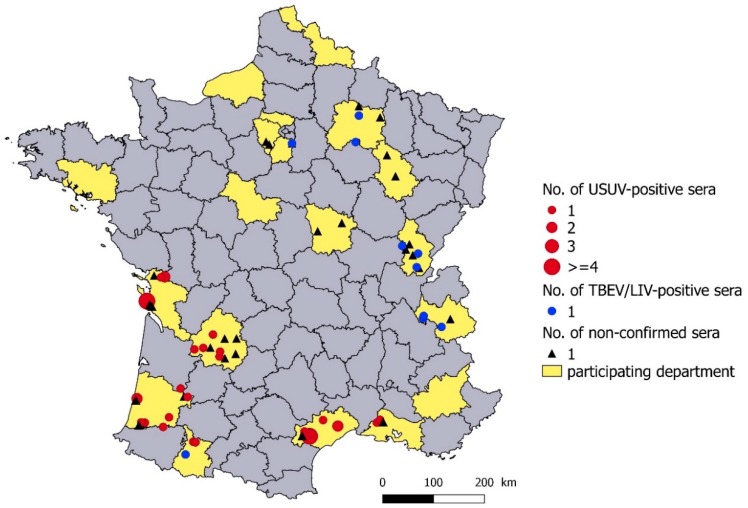
Geographical distribution of USUV-positive and TBEV/LIV-positive serum samples by virus micro-neutralization test (MNT) or xMAP microsphere immunoassay (MIA), and unidentified flavivirus-positive serum samples in cELISA and negative or not tested by MNT and MIA. The number represents the number of serum samples per municipality.

USUV antibodies were detected in animals from six departments, located in southwestern and southeastern France by MNT and/or MIA (Figure 2). TBEV/LIV MNT- and MIA-positive antibodies were detected in three departments of eastern France (Figure 2). TBEV/LIV antibodies were detected only by MIA in three departments located in eastern (Marne, department 51), southwestern (Hautes-Pyrénées, department 65) and northern France (Yvelines, department 78) (Figure 2, Table 1). The 28 serum samples found to be positive or doubtful with the pan-flavivirus cELISA that could not be tested (low serum quantity or too hemolysed) or confirmed by MIA and MNT were from 11 departments (Figure 2). They included nine departments in which USUV or TBEV/LIV antibodies had been confirmed, and two from eastern France close to the TBEV endemic area. No flavivirus antibodies were detected in four departments with >30 serum samples tested: Three were located in western and northern France and one was located in far southeastern France. 

### 3.3. Seroprevalence in USUV-Infected Departments 

In USUV-infected departments, an apparent pan-flavivirus seroprevalence of 10.0% (CI_95%_: 7.3–13.2%) was obtained in wild boar and 3.1% (CI_95%_: 1.1–6.5%) in roe deer. The USUV seroprevalence detected by MNT and MIA was 8.0% (CI_95%_: 5.6–11.0%) in wild boar and 1.0% (CI_95%_: 0.1–3.6%) in roe deer. None of the juvenile roe deer (*n* = 38) were found to be seropositive. Five juvenile wild boar sampled in 2012 and 2013 had USUV antibodies. The USUV seroprevalence was higher in adults (12.3%, CI_95%_: 8.8–16.5%) than in juveniles (4.3%, CI_95%_: 1.4–9.7%). In adults, the USUV seroprevalence increased linearly with the period (*p* = 0.02, Wald test) from 2.6% (CI_95%_: 0.3–9.1%) in 2009–2011 to 12.6% (CI_95%_: 7.3–19.6%) in 2013–2015. The GLMM showed that the probability of being USUV-seropositive was higher for wild boar adults and animals sampled in 2013–2014 and 2014–2015 compared to 2009–2010 (Table 3).

### 3.4. Seroprevalence in TBEV/LIV-Infected Departments in Eastern France

In TBEV/LIV-infected departments in eastern France, an apparent pan-flavivirus seroprevalence of 2.9% (CI_95%_: 1.2–5.9%) was obtained in wild boar, and 1.9% (CI_95%_: 0.7–4.0%) in roe deer. The TBEV/LIV seroprevalence detected by MNT and MIA was 2.9% (CI_95%_: 1.2–5.9%) in wild boar and 0.6% (CI_95%_: 0.1–2.2%) in roe deer. None of the juveniles (*n* = 59 wild boar and 91 roe deer) were TBEV/LIV-seropositive. TBEV/LIV antibodies have been detected annually from 2010 to 2014. According to the GLMM results, species, age class and sampling period were not associated with the probability of an animal being TBEV/LIV-seropositive. 

## 4. Discussion

We used wild ungulate serum samples collected from 2009 to 2014 to investigate past circulation of flaviviruses in France. USUV and TBEV/LIV antibodies were detected in roe deer and wild boar. 

### 4.1. Spatiotemporal Circulation of USUV in France

Our results highlighted USUV circulation in southwestern and southeastern France from s2009 to 2014. These results confirm long-term USUV circulation in southeastern France, as reported earlier in 2009–2010 by Vittecoq et al. [15], with four common magpies (*Pica pica*) in the Camargue area displaying USUV-neutralizing antibodies, while USUV infection could not be established firmly in two additional animals presenting USUV- and WNV-neutralizing responses. Our results support the hypothesis of continuous circulation of USUV in the study period, considering that USUV seroprevalence tended to increase over the study period and was higher in adult wild boar than in juveniles. USUV antibodies were detected every year from 2009 to 2014 in adults, and only in 2012–2013 and 2013–2014 in wild boar juveniles, likely to be older than six months. There are no data on the persistence of maternal USUV antibodies in wild boar. Considering that maternal antibodies against most viruses disappear after six months in large mammals [41], our results would suggest that USUV circulated at least in summer and autumn of 2012 and 2013. Although flavivirus antibody persistence in artiodactyl species is unknown, a longer time span of exposure and antibody persistence could be associated with the higher seroprevalence found in adults, as suggested by field observations for WNV [31,34,35,37]. Given the potential long persistence of antibodies in artiodactyls, it is impossible to know from our results whether USUV circulated every year since 2009 or was re-introduced in 2012. 

Very few studies have been conducted to assess exposure of wild ungulates to USUV in infected areas in Europe. In these studies, USUV antibodies in wild ungulates were detected by MNTs to exclude cross-reactivity with WNV antibodies in areas endemic for WNV. The prevalence of neutralizing antibodies against USUV detected in wild boar (8%) and in roe deer (1%) in the infected departments studied here was slightly higher than that found in two studies conducted in endemic WNV countries: In Serbia in 2010–2011 (3.4%, *n* = 318 wild boar, 0%, *n* = 91 roe deer), and in Spain from 2003 to 2014 (0.2%, *n* = 4335 red deer, 0%, *n* = 32 roe deer) [34,42]. However, this apparent difference may only arise from the fact that the authors estimated the seroprevalence in both infected and uninfected areas, contrary to our results. Similar seroprevalence values in wild ungulates have been observed for WNV antibodies in endemic WNV areas in Serbia, Spain and the Czech Republic. This seroprevalence varied from 2% to 13% in wild boar [31,35,42,43,44], and from 4% to 15% in roe deer [42,43,44]. 

In our study, wild boar showed higher seroprevalence for USUV than roe deer. Among the seropositive animals by cELISA, the confirmatory rate by MNT and MIA was higher and the mean % S/N of cELISA was lower for wild boar, suggesting higher antibody titrer in wild boar. Similar differences in the seroprevalence of flavivirus infections (as regards WNV) were found between wild boar and cervids (roe deer and red deer) in Spain [28,31,35]. These results may suggest lower antibody response in roe deer to infections by flaviviruses of the Japanese encephalitis antigenic complex. Alternatively, the observed difference may arise from lower exposure of roe deer to mosquito vector bites related to a difference in terms of animal size, habitat or feeding preferences of *Culex* mosquitoes between wild boar and roe deer. 

Surprisingly, USUV was not detected in other departments of eastern and central France in our study, whereas USUV has frequently been detected in these areas from 2015 to 2018, mainly after unusual and grouped bird fatalities [13,16,17,18]. The year 2018 was marked by a sudden increase in the reporting of USUV outbreaks, with a massive die-off of blackbirds (*Turdus merula*) and captive owls (*Strix nebulosa* and *Bubo scandiacus*) in most parts of France [13,18]. From 2015 to 2018, three lineages of USUV were detected, suggesting multiple introductions in France [13,16,17,19]. A Europe 3 strain, genetically close to strains circulating in Eastern Europe since 2011 (Germany, Czech Republic), was detected in dead birds from 2015 to 2017 in northeastern France (Alsace-Lorraine) [16]. Strains from the Africa 3 lineage, genetically close to the strain circulating in Germany in 2014 and in Belgium and the Netherlands in 2016 [9], were detected in central and southern France from mosquito pools in 2015 [17] and from birds in 2018 (Lecollinet, pers. comm). The Africa 2 lineage was detected on several occasions in 2015–2016 in central France (Rhône department) after bird mortalities, as well as in southern France on mosquito pools, and from a human patient suffering from a neurological disorder [16,17,19]. This lineage was previously identified on two occasions in Spain, in 2006 and 2009, from *Culex* mosquito samples, but was not associated with bird morbidity before its identification in European countries further east, such as France and Germany in 2015 [16,45,46,47,48]. It is impossible to infer from our results which USUV strains were circulating from 2009 to 2014 in southern France. Importantly, USUV circulation remained undetected by the French event-based surveillance network SAGIR [49] that monitors abnormal wild bird mortalities. Since their first detection in Europe, the USUV Europe 3 and Africa 3 lineages have been responsible for massive die-offs of song birds in several countries [9,16,43,50,51,52,53,54]. On the contrary, given that limited cases of bird mortality due to the Africa 2 lineage have been reported, this lineage appears to be less pathogenic for birds. Therefore, we hypothesize that USUV circulating in southern France from 2009 to 2014 may belong to the Africa 2 lineage, close to the strains found in Spain in 2006 and 2009 and in southeastern France (Bouches-du-Rhône) in 2015 [17,47,48]. The absence of detection of USUV antibodies in eastern and central France in our study suggests that both USUV lineages Europe 3 and Africa 3 were introduced more recently than 2014. 

### 4.2. No Detection of WNV and BAGV Antibodies 

No WNV antibodies were detected in wild ungulates sampled from the known endemic areas for WNV in southeastern France, the two departments Bouches-du-Rhône (department 13, Figure 1C) and Hérault (department 34, Figure 1C), whereas Vittecoq et al. [15] found WNV antibodies in a second-year common magpie in 2010 in the Bouches-du-Rhône, suggesting recent WNV infection (in 2009 or 2010) in the area. In southeastern France, WNV has been reported irregularly and is generally detected when the virus circulation is high and causes clinical outbreaks in humans and horses. Such epizootics in the Camargue area have been observed in 2000, 2004, 2015 and 2018 [11,13,55,56]. Between two epizootics, the virus could be silently maintained, as suggested by the detection of WNV antibodies in wild birds [15,57]. In our study, few animals were sampled in areas located close to the wetlands of the Hérault and Bouches-du-Rhône departments, where WNV cases were mostly detected during the outbreaks. This may have limited the detection of WNV circulation in our study. This result confirms that the virus, if present, probably circulates very locally close to wetland areas that provide sustainable habitats for the nesting of many wild resident or migrating birds, and for the multiplication of *Culex* vectors [58]. 

Bagaza virus antibodies were not detected. In Europe, this virus was only detected in southern Spain in 2010 and 2011, associated with high mortality of red-legged partridges (*Alectoris rufa*) and ring-necked pheasants (*Phasianus colchicus*) [59,60]. The main hosts of BAGV are birds and there is little evidence of BAGV antibodies in mammals. Neutralizing antibodies to BAGV have been found in serum samples from encephalitis cases in humans in India, and in experimentally infected mice [61,62]. 

### 4.3. TBEV Serocomplex Distribution

We detected TBEV/LIV antibodies in three departments of eastern France (Savoie department 73, Marne department 51, and Jura department 39 in Figure 1C), in one department in the south (Hautes-Pyrénées, department 65) and one department in northern France (Yvelines, department 78). Given the genetic and antigenic relatedness between TBEV and LIV, serological responses induced after TBEV and LIV infection could not be differentiated by MNT and MIA. Although the distribution of TBEV in France is not known precisely and clinical cases have mainly been reported in the far eastern parts of France (Alsace-Lorraine and Alpine regions), the virus is thought to be broadly distributed in the east of France. We detected TBEV/LIV antibodies in wild ungulates in one department (Savoie), where TBE clinical cases in humans have been reported [20], and in two departments (Marne and Jura), where forest workers were previously found to be seropositive to TBEV [22]. It is therefore likely that seropositive wild ungulates observed in our study in eastern France have been exposed to TBEV. 

The wild boar found to be TBEV/LIV-seropositive in the Hautes-Pyrénées department by MIA was not confirmed by MNT, whereas the serum was strongly positive in cELISA (low mean % S/N). For the wild boar in the Yvelines department, the serum was too hemolysed and the result could not be confirmed by MNT. The result obtained by MIA could be interpreted, with caution, as an indicator of a tick-borne flavivirus infection. TBEV/LIV seropositivity in Hautes-Pyrénées could originate from an infection with LIV or LI-like virus, since these viruses have been reported in sheep, goats and Cantabrian chamois (*Rupicapra pyrenaica parva*) in close geographical areas in Spain: In the Basque and Asturias regions [23,25,63]. The presence of a virus from the TBEV-serocomplex was found at a broader scale in Spain by serology, with antibodies neutralizing TBEV reported in southern Spain and on the island of Mallorca [28,29]. The positive serum in the Yvelines might also reveal the western limit of distribution of TBEV in France. Interestingly, TBEV was recently detected in ticks in the United Kingdom, which represents the western-most distribution limit for TBEV [64]. Finally, in both cases, we cannot rule of the hypothesis that this might be due to another tick-borne flavivirus. The diversity and distribution of tick-borne flaviviruses are not well known, and new species are regularly discovered in ticks [65]. Further investigations are needed to confirm the circulation of another tick-borne flavivirus in France and to isolate this virus. 

The overall TBEV seroprevalence found in wild boar and roe deer in the eastern departments (2.9% in wild boar and 0.3% in roe deer) was much lower than that found in TBEV endemic areas in Germany, Poland, Slovakia, the Czech Republic and the south of Sweden, varying from 10% to 40% [32,36,44,66,67,68,69]. However, it was similar to the seroprevalences found in areas with the same epidemiological situation as eastern France, with no or very few human clinical cases: From 2% to 6% seropositive wild boar and roe deer have been reported in Belgium, in some areas of Germany, and in the south of Denmark [66,70,71,72,73]. We did not find any significant difference in TBEV seroprevalence between wild boar and roe deer, which is consistent with previous observations [44,66,69,71,72]. These results are unexpected as roe deer are considered to be the main hosts for the nymphs and adults of *Ixodes ricinus*, the main tick vector for TBEV in Western Europe [74]. However, there is a lack of data on the role of wild boar as a feeding host for this tick or for other potential vector tick species of TBEV, such as *Dermacentor* spp. [75] 

### 4.4. Unidentified Flaviviruses 

Twenty-seven samples were positive or doubtful according to cELISA and negative according to MNT and MIA for WNV, USUV, TBEV, LIV and BAGV, or negative according to MIA and not tested by MNT. All but four samples were located in departments where USUV or TBEV/LIV antibodies had been detected in our study. The four other samples were located in two eastern departments, where TBEV antibodies had previously been detected in forest workers [22]. Three samples were located in the Yvelines (department 78), where antibodies against TBEV/LIV were detected in one serum sample. We cannot exclude the possibility that another flavivirus may be circulating. However, given their localization, it is more likely that the animals were exposed to USUV or TBEV. Most probably, ELISA positive or doubtful results could not be confirmed by MNT or MIA due to serum quality or the lower analytical sensitivity of these tests, which is consistent with the low antibody titres (high % S/N) found by cELISA for most of these serum samples. 

## 5. Conclusions

The results obtained in this study documented the distribution and spatiotemporal dynamics of flaviviruses in France from 2009 to 2014. They indicate silent and probable continuous circulation of USUV in southwestern and southeastern France from 2009 to 2014. Antibodies against TBEV/LIV were detected in two eastern departments, where no TBE clinical cases have been reported so far, a finding that confirms the wider distribution of the TBE virus in the east of France. The serum samples with TBEV/LIV antibodies found in southwestern and northern France warrant further exploration to determine the virus responsible for the seroconversion. This study shows the usefulness of serum banking by hunters concerning wildlife for retrospective epidemiological surveys of pathogen circulation. 

## Figures and Tables

**Table 1:** Serological results on wild boar and roe deer serum samples tested by pan-flavivirus competitive ELISA, virus micro-neutralization tests (MNTs) and xMAP microsphere immunoassays (MIAs) by department (Dpt) (see Figure 1 for the geographical localization). Pos.: Positive, Dbt; doubtful, **^§^** proportion of positive results by MNT or MIA among all the samples tested by cELISA; ^a^ three and ^b^ four serum samples not tested by MNTs.

Dpt	Wild Boar									Roe Deer								
	No. of Samples	cELISA		USUV-Positive			TBEV/LIV-Positive			No. of Samples	cELISA		USUV-Positive			TBEV/LIV-Positive		
		Pos. or Dbt	%	MNT	MNT or MIA	% ^§^	MNT	MNT or MIA	% ^§^		Pos. or Dbt	%	MNT	MNT or MIA	% ^§^	MNT	MNT or MIA	% ^§^
4	38	0	0%	-	-	0	-	-	0%	40	0	0%	-	-	0%	-	-	0%
13	34	3	8.8%	2	2	5.9%	0	0	0%	2	0	0%	-	-	0%	-	-	0%
17	50	10	20.0%	8	9	18.0%	0	0	0%	44	2	4.5%	0	0	0%	0	0	0%
24	61	7 ^a^	11.5%	2	3	4.9%	0	0	0%	60	3	5%	2	2	3.3%	0	0	0%
34	212	11	5.2%	9	10	4.7%	0	0	0%	19	0	0%	-	-	0%	-	-	0%
39	128	3	2.3%	0	0	0%	1	2	1.6%	176	4	2.3%	0	0	0%	0	1	0.6%
40	30	10	33.3%	7	8	26.7%	0	0	0%	31	1	3.2%	0	0	0%	0	0	0%
41	8	0	0%	-	-	0%	-	-	0%	-	-	-	-	-	-	-	-	-
51	26	2	7.7%	0	0	0%	0	2	7.7%	54	2	3.7%	0	0	0%	0	0	0%
52	127	1	0.8%	0	0	0%	0	0	0%	85	1	1.2%	0	0	0%	0	0	0%
56	-	-	-	-	-	-	-	-	-	33	0	0%	-	-	0%	-	-	0%
58	26	0	0%	-	-	0%	-	-	0%	25	2	8.0%	0	0	0%	0	0	0%
59	29	0	0%	-	-	0%	-	-	0%	22	0	0%	-	-	0%	-	-	0%
65	37	3	8.1%	2	2	5.4%	0	1	2.7%	40	0	0%	-	-	0%	-	-	0%
73	85	3	3.5%	0	0	0%	3	3	3.5%	90	1	1.1%	0	0	0%	0	0	0%
76	29	0	0%	-	-	0%	-	-	0%	37	0	0%	-	-	0%	-	-	0%
78–91–95	94	4 ^b^	4.2%	0	0	0%	0	1	1.1%	-	-	-	-	-	-	-	-	-
TOTAL	1014	57	5.6%	30	34	3.4%	4	9	0.9%	758	16	2.1%	2	2	0.3%	0	1	0.1%

**Table 2:** Contingency table of and xMAP microsphere immunoassays (MIAs) and virus micro-neutralization tests (MNTs) results for 66 samples revealed as positive or doubtful by competitive ELISA and tested by both MIAs and MNTs.

Confirmation Method		MIA			Total
		Positive for USUV	Positive for TBEV/LIV	Negative	
MNT	Positive for USUV	26	0	6	32
	Positive for TBEV/LIV	0	1	3	4
	Negative	3	5	22	30
Total		29	6	31	66

**Table 3:** Results of the generalized linear mixed model (logistic link function) of USUV seropositivity in wild ungulates in southern France, with the department included as random factor. OR: Odds ratio, 95% CI: 95% confidence intervals estimated by bootstrap.

Variables	No. of Individuals	No. of Positives	β	Error	OR	95% CI Bootstrap	*p*
**Species**							
roe deer	196	2	a	a	a	a	a
wild boar	419	34	2.66	0.66	17.3	4.8–9.6.10^8^	<0.001
**Age Category**							
adult	458	31	a	a	a	a	a
juvenile	157	5	−1.28	0.52	0.3	0.1–0.6	0.01
**Sampling Period**							
2009–2010	62	2	a	a	a	a	a
2010–2011	81	1	−1.01	1.23	0.4	0–75.2	0.41
2011–2012	88	4	0.19	0.9	1.2	0.1–1.5.10^8^	0.83
2012–2013	198	12	1.08	0.83	2.9	0.9–7.3.10^8^	0.19
2013–2014	156	13	1.84	0.82	6.3	1.5–1.3.10^8^	0.02
2014–2015	22	4	3.17	1.04	23.9	3.9–5.3.10^8^	0.002

a: reference category.
